# An Exploratory Investigation of Representations of Herpes Zoster and Adjuvanted Recombinant Herpes Zoster Vaccination in a Sample of Fragile Adults in Italy

**DOI:** 10.3390/pathogens14020145

**Published:** 2025-02-04

**Authors:** Francesco De Caro, Nadia Pecoraro, Mario Capunzo, Simona Caruccio, Filippo Caggiano, Giuseppina Cersosimo, Maria Costantino, Walter Longanella, Francesca Malatesta, Matteo Tomeo, Giulia Savarese, Pio Sinopoli, Emilia Anna Vozzella, Giuseppina Moccia

**Affiliations:** 1 Public Health Laboratory for the Analysis of Community Health Needs, Department of Medicine, Surgery and Dentistry, University of Salerno, Baronissi Campus, 84081 Baronissi, Italy; fdecaro@unisa.it (F.D.C.); npecoraro@unisa.it (N.P.); 2 Department of Medicine, Surgery and Dentistry, University of Salerno, Baronissi Campus, 84081 Baronissi, Italy; mcapunzo@unisa.it (M.C.); scaruccio@unisa.it (S.C.); mcostantino@unisa.it (M.C.); mtomeo@unisa.it (M.T.); gsavarese@unisa.it (G.S.); psinopoli@unisa.it (P.S.); 3 Hospital San Giovanni di Dio e Ruggi d’Aragona, 84131 Salerno, Italy; filippo.caggiano@sangiovannieruggi.it (F.C.); walter.longanella@sangiovannieruggi.it (W.L.); direzione.sanitaria@sangiovannieruggi.it (E.A.V.); 4 Department of Political and Social Studies, University of Salerno, 84084 Fisciano, Italy; gcersosi@unisa.it

**Keywords:** Herpes Zoster, illness representations, vaccination, hospital vaccination, fragile patients, vaccinal campaigns, vaccine hesitancy

## Abstract

In the context of the Italian National Herpes Zoster Vaccine program, an exploratory survey was conducted on a sample of fragile adult patients to investigate the representations of the disease and its prevention to build future local vaccination campaigns. An ad hoc questionnaire was administered to 271 fragile adult patients who had adjuvanted recombinant Herpes Zoster vaccination to detect the following: knowledge and perception of the disease and its risks; information sources and confidence in the information sources used; and perception of the Herpes Zoster vaccination. Fragile adult patients have the representation of Herpes Zoster as a serious disease (86.5%), and they consider themselves informed concerning symptoms and health effects. Women are more fearful of the impact of the disease (Chi-square = 10.03; DF = 3; *p*-value = 0.018), while those with a higher average age consider themselves less informed (R = −158; *p*-value = 0.039). The sources of information that contributed to the construction of illness representation are health personnel (73.5%), followed by the web and social web (14.7%), and media such as radio and TV (10.0%). Regarding the vaccine representation, fragile patients are confident about the vaccine and the science behind it and believe everyone should receive it. However, a high percentage (62.9%) fear side effects. Our analysis highlights that vaccination campaigns must be planned based on the target audience, individual and contextual needs, and representations of the disease, particularly when dealing with frail patients, to implement effective preventive interventions.

## 1. Introduction

Herpes Zoster (HZ) is a viral disease caused by reactivation of varicella-zoster virus (VZV) that can occur in concomitance with periods of mental and physical stress, immunosuppressive drug therapies, diseases affecting the immune system, or in fragile individuals with compromised immune defenses [1,2,3]. This condition mostly affects people over 60 years old, and its average duration is about 5–6 months, but it may persist months or even years after the rash has healed [4,5]. Research has shown that age is the main risk factor for onset and severity and that HZ has a more significant and lasting impact on frail patients with previous chronic conditions [6,7].

For a long time, the standard vaccine for HZ prevention has been a live attenuated virus vaccine [8,9]. In 2017, a new recombinant zoster vaccine (RZV), based on adjuvanted glycoproteins, was approved and it was recently included in the Italian National Vaccine Prevention Plan. This vaccine is indicated in individuals older than 50 years and frail individuals older than 18 years at increased risk of HZ [2,10]. It represents, therefore, a significant advance in the prevention of HZ reactivation in frail individuals [11]. RZV is 97.2% effective in adults aged 50 years and older and 91.3% effective in those aged 70 years and older; its efficacy remains above 90% in adults aged 70 years and older. In addition, RZV has demonstrated high efficacy (87.2%) even in immunocompromised individuals, such as those with hematologic malignancies, where other vaccines are generally less effective [12,13]. RZV is associated with an incidence of adverse events that are typically mild to moderate in severity and usually resolve within a few days, such as local reactions at the injection site, muscle pain, and symptoms such as fever and fatigue [14].

Vaccination is an effective tool to reduce the incidence and severity of HZ and to provide protection against post-herpetic neuralgia (PHN), which is the most common chronic complication of HZ, contributing to an improvement in living conditions [4,15,16].

In 2023, the systematic review conducted by Wang et al. found that the aggregate HZ vaccination readiness rate is only 55.74% worldwide [17], while the World Health Organization (WHO) has found that the willingness to receive HZ vaccines of populations older than 50 years does not exceed 50% [18,19,20]. The main reasons related to reluctance to receive the HZ vaccine include a low perception of disease risk, low confidence in vaccine efficacy and safety, and lack of knowledge about the vaccine availability [17,21]. Studies have shown that people’s attitudes, beliefs and emotions about a disease and its vaccine can influence their intention to vaccinate, as occurred in the case of COVID-19 [22,23,24,25,26]; this occurs, in particular, in the case of patients with chronic conditions.

In general, the decision to vaccinate is influenced by multiple factors: sociodemographic, cognitive, psychological, socio-cultural, and political and organizational [27,28,29,30].

There is good evidence that vaccine uptake is shaped by sociostructural forces. These forces not only produce different worldviews, but also present different vaccination opportunities and availability, promote different information and knowledge about vaccination, and create different priorities in managing a life at risk.

Social and psychological research [31,32,33] has highlighted the fundamental role of representations of health and illness through which social groups interpret phenomena and organize related reality. This is the case for diseases and practices of treatment and prevention [34]. Representations of health and illness have an interpersonal/collective level, related to social representation [33,35,36], and an intrapsychic level related to individual representation that is co-constructed with social representation [37]. The levels of social representations consist of a central core, representing the common basis of collective memory, and a peripheral system that integrates central information with local and individual practices and experiences [26].

According to Karoly’s model [38], the individual constructs their reality of illness through two processes of self-regulation, one cognitive and one emotional. These processes guide the individual in taking an active role in the process of collection and interpretation of information from various sources, determining, then, the activation of health behaviors [39,40]. The person, in constructing their representation, uses and integrates information already assimilated from cultural knowledge about the disease with information based on current or previous experience of the disease and the outcomes of previously adopted coping strategies. To these, finally, is added information received from significant or authoritative people in the external social environment [41,42]. Typically, the cognitive representation of the disease is divided into interrelated dimensions: disease identity, which provides a framework for different emotional and behavioral reactions; causal dimension; duration; consequences; and controllability or curability (the perceived sense of power concerning the adoption of successful coping strategies or the effectiveness of prescribed treatment) [43].

The purpose of this study is to identify the representations of HZ and vaccination that influenced pro-vaccination behavior concerning HZ in a sample of frail adult patients and to identify dimensions and interpretive areas on which to reflect for the construction of future vaccination campaigns in specific patient samples.

This activity aims to conduct an exploratory survey to detect dimensions regarding (a) knowledge and perception of HZ disease and its associated risks; (b) information sources on HZ and confidence in information sources; and (c) perception of the Herpes Zoster vaccination.

## 2. Materials and Methods

### 2.1. Procedure

A vaccination project against HZ aimed at frail adult patients was implemented at the University Hospital “San Giovanni di Dio e Ruggi d’Aragona”, Salerno (Campania, Italy). The vaccine project included the drafting of an application protocol, starting with ministerial recommendations regarding the HZ vaccine program, that is, the National Vaccine Prevention Plan (PNPV) for 2023–2025, approved in the State-Regions Conference on 2 August 2023. The Italian Ministry of Health stipulates that the Herpes Zoster vaccination should be actively offered to individuals aged 65 years and those with pre-existing frail conditions, such as hypertension, heart problems, diabetes, chronic respiratory diseases, and oncological or on hematological diseases, as well as patients undergoing dialysis treatment, transplanted patients or those awaiting organ transplantation, and immunocompromised patients (due to congenital or acquired diseases, transplants or being treated with immunosuppressive drugs) (Circular no. 008770 dated 8 March 2021). Although not mandatory, vaccination has been strongly recommended nationwide and is provided free of charge to the groups mentioned above. At the regional level, Italy’s decentralized healthcare system, which includes the administrative autonomy of regional governments, enters into agreements with healthcare partners (e.g., general practitioners (GPs), pharmacies and nursing homes, and medical personnel) that adhere to the immunization campaign to implement adherence. Specifically, university hospitals are not institutionally required to conduct a needs analysis and vaccination; this is an additional strategy to that of the Local Health Authority, which is institutionally required to perform this function.

The university hospital “San Giovanni di Dio e Ruggi d’Aragona” is a Level II Department of Emergency and Acceptance (DEA), which has about 120,000 accesses annually. The methodology tested at this hospital frames the vaccine as an additional drug within the care plan and aims to avoid complications during the hospitalization period and subsequently to protect the health status of the frail patient. The goal of the research is to implement new immunization strategies to build new vaccine campaigns in the future.

In this case, the departments that joined the vaccination campaign were as follows: Nephrology-Dialysis and Kidney Transplantation Unit, Hematology Unit and Bone Marrow Transplant Center, Infectious Diseases Unit, and Gastroenterology Unit of the “Ruggi” Hospital of Salerno (Campania, Italy); and the Dialysis Unit of the “A. Fucito” Hospital of Mercato San Severino, Salerno (Campania, Italy).

The method implemented included the involvement of specific operating units to identify frail adult patients through training of and information for healthcare personnel; patient involvement with specific information on the HZ virus, vaccine vaccine type, and vaccination strategy; and planning and implementation of a traveling vaccination program, that is a mobile vaccination unit (equipped with a portable refrigerator so as not to interrupt the cold chain), which followed the patient’s care pathway in the referral ward during the first visit or follow-up.

Vaccination of frail adult patients was carried out in the hospital between December 2022 and March 2024. The recruited patients underwent an anti-HZ vaccine cycle with adjuvanted recombinant vaccine (Shingrix-GSK). After the RZV, patients reported at least one adverse event following immunization (AEFIs), particularly frail patients aged 51 to 60 years: pain at the injection site followed by fever; local skin reactions such as rash, swelling and itching; and exhaustion-like fatigue. There was no dropout rate as all recruited patients adhered to the questionnaire administration and received the HZ vaccine.

### 2.2. Tools and Data Collection

The survey was conducted using an ad hoc questionnaire created based on the literature review and expertise of the specialists involved in the present study. The questionnaire was entered on the Google Forms platform via a QR code. Participants were asked to complete this questionnaire at the vaccination stage, which comprised the following sections:

(1) Informed Consent Form and Privacy Policy, with which participants were informed of the purpose of the research and provided informed consent to the use of their data in an anonymous and aggregate form, in accordance with the General Data Protection Regulation of the European Union. This section included information on the vaccine to be administered (injection site, lot number, expiration date, route of administration, and health professional data);
(2) Individual data collection, including demographic information (sex, marital status, occupation), education level, presence of pathologies, previous vaccinations, and previous presence of HZ;
(3) An ad hoc scale was created for the survey. The scale consists of 17 items with multiple-choice questions. The dimensions explored were as follows:
  - Knowledge and perception of Herpes Zoster disease and its associated risks;
  - Information sources on HZ and confidence in information sources. Several areas were assessed: the type of information sources used to gain knowledge about the disease, and the degree of satisfaction regarding HZ information and awareness campaigns carried out at national and local levels;
  - Perception of the Herpes Zoster vaccination. This area assesses an individual’s favorable or unfavorable stance toward vaccination and the risk and protective factors that may influence it.

### 2.3. Statistical Analysis

Data collection and statistical analysis were carried out using IBM SPSS v.28 software (IBM^®^ SPSS^®^, Bologna, Italy).

A descriptive analysis was conducted to measure the socio-demographic variables of the sample. A frequency analysis was conducted on the items concerning the three dimensions explored.

An analysis was then carried out, as well as inferential statistics, by cross-referencing the results obtained with the socio-demographic variables.

## 3. Results

### 3.1. Participants

A total of 271 frail adult patients took part in this survey (F = 37.7%; M = 62.3%; mean age = 55, SD = 13.6). Women had a mean age of 52 years (SD = 16), and men 57 years (SD = 12).

Regarding marital status, 67.6% of the participants were married, 6.5% were separated/divorced, 19.4% were single, 3.0% were widowed, and 3.5% were cohabiting.

The level of schooling was as follows: 77.6% had completed secondary education, while the remaining 22.4% had at least college-level or higher (post-graduate training). As for occupation, 14.1% were office workers, 4.1% were teachers, 11.8% were homemakers, 11.8% performed laborer/craftsman work, 10.6% were self-employed, 2.4% were students, 28.2% were retired, 8.2% were unemployed, 8.2% stated “other”.

Regarding contact with the Herpes Zoster, 25.0% of the participants had Herpes Zoster, while 75.0% had never (Table 1).

Regarding the diseases represented among all the responses, 17.5% reported chronic heart disease, 63.3% had hypertension, 21.1% had diabetes mellitus, 7.8% had pulmonary diseases, 77.1% were following immunosuppressive therapy, 7.2% had rheumatological diseases, 6.6% had oncological diseases, 1.8% were undergoing dialysis treatment, 2.4% were awaiting organ transplantation, and 4.8% had hematopoietic stem cell transplantation (Table 2).

Regarding vaccines, out of the total of responses, 98.2% had taken the COVID-19 vaccine, 50.6% had taken the flu vaccine, and finally, 2.9% had taken other vaccines.

### 3.2. Quantitative Analysis

The results of each of the areas investigated will be presented in order, reporting analyses of the percentage frequencies related to the items and inferential statistics analyses with respect to the relationship between them:

(a) Knowledge and perception of Herpes Zoster disease and its associated risks

On the item “I am sufficiently informed about the Herpes Zoster virus”, patients showed high percentages of agreement (“partially agree” (41.8%) and “totally agree” (42.9%), while 8.8% reported they “partially disagree”, and 6.5% chose “totally disagree” (Table 3).

Concerning the item “I am adequately informed about the symptoms and consequences of the Herpes Zoster virus”, 41.8% “partially agree”, while 40.6% “totally agree”, 11.8% stated they “partially disagree”, and 5.9% chose “totally disagree”.

The degree of agreement decreases with increasing age (R = −158; *p*-value = 0.039), that is, the perceived knowledge of the symptoms and consequences of the virus decreases with age.

When asked, “Herpes Zoster is a severe disease”, 44.7% stated they “partially agree” and 41.8% “totally agree” with this statement; 10.0% “partially disagree” and 3.5% “totally disagree”. As age increases, differentiated positions are expressed. Those who “totally disagree” with the statement have an average age of 61.2 years (SD = 12.5), and those who “totally agree” are 57.7 years (SD = 13.75). In the middle positions are those who “partially agree” (Mean = 53.6; SD = 13.5) and “partially disagree” (Mean = 48; SD = 10.55), (F = 3.203; DF = 3; *p*-value = 0.025).

Concerning the risk perception of the effects of the disease, based on the question “I do not fear the effects of Herpes Zoster”, the sum of agreement positions was 53.5%, while that of the disagreement positions was 46.4%. Comparing the expected frequencies with those obtained shows that women are more likely to disagree with this statement than men (Chi-square = 10.03; DF = 3; *p*-value = 0.018) (Table 3).

(b) Information sources on HZ and confidence in information sources

To the item “What are the major sources of information used?” participants answered as follows: media (TV, radio), 10.0%; web and social web, 14.7%; medical personnel, 73.5%; and finally, word-of-mouth, 1.8% (Table 4).

To the question “In your experience, how satisfied are you with the vaccine information and awareness campaigns implemented by the Italian government?”, 58.9% reported they were “fairly satisfied” or “satisfied”, 24.1% were “fairly dissatisfied” or “dissatisfied”, 14.1% were neutral, and 2.9% said they had no experience with them.

Regarding satisfaction with government information campaigns, those with postgraduate education lean toward being more dissatisfied (29.9% were either “quite dissatisfied” or “dissatisfied”) or are in the neutral area (18.4% were “neither satisfied nor dissatisfied”); however, overall, they remain at 50.8% satisfied. In contrast, it emerges that those with a lower degree of education lean toward lower dissatisfaction (22.8% were “quite dissatisfied” or “dissatisfied”); in the neutral area, 12.9% were “neither satisfied nor dissatisfied”, while 60.6% reported they were “satisfied”; and 3.8% “had no experience with this” (Chi-square = 11.58; DF = 5; *p*-value = 0.041).

Concerning HZ and vaccine information, for the item “I have confidence in the information I have received from the medical staff”, participants reported that they confidently trust medical personnel, with 66.5% “totally agreeing” with the statement and 25.9% “partially agreeing”. The correlation is negative with age (R = −166; *p*-value = 0.030), that is, trust decreases with increasing age.

The confidence concerning the information acquired from the media, although high, is lower than that acquired from medical personnel. When asked, “I have confidence in the information I acquired from the media”, only 27.0% responded to the statement with “totally agree”, 45.9% with “partially agree”, 7.1% with “totally disagree”, and the remaining 20.0% were “partially agree”. In addition, there is a positive correlation between age and this item (R = 0.176; *p*-value = 0.22).

Regarding confidence about the information received from family members, when asked, “I have confidence in the information I have acquired from my family members”, 87.1% stated they “totally agreed” or “partially agreed”, 4.7% reported they “totally disagreed”, and the remaining 8.2% “partially disagreed” (Table 4).

Inferential statistics analysis shows that afference to sources differed by gender (Chi-square = 9.506; DF = 3; *p*-value = 0.023). Men use doctors’ information more (79.2%), while women rely on different sources, with a greater focus on media (TV, radio) than men (women = 17.2%; men = 5.7%) and the social web (women = 18.8%; men = 12.3%). Women do not rely on word-of-mouth (Chi-square = 9.50; DF = 3; *p*-value = 0.023) (Table 5).

Another relevant result concerns the relationship between profession and access to sources (Table 5). Teachers use the web and social web (42.9%) and media (TV, radio) (28.6%) as their source of information the most and proportionally less than doctors, while the other professional categories rely more on the role of the doctor (Chi-square = 46.46; DF = 24; *p*-value = 0.004) compared with information from the web and social web (Table 5).

Regarding diseases, those with chronic heart disease say they informed themselves partly through the media (TV, radio) (24.1%) and through the web and social web (10.3%), as well as through physicians (65.5%) (Chi-square = 8.36; DF = 6; *p*-value = 0.039).

Cross-tabulation of information about the HZ virus with the degree of satisfaction with the government vaccination campaign shows that the degree of satisfaction with government vaccination campaigns above a threshold level of 50 percent corresponds to higher agreement on the degree of information about the disease.

In other words, those who feel sufficiently informed about the virus are satisfied with government vaccination campaigns (Chi-square = 27.071; DF = 15; *p*-value = 0.028).

Still considering the degree of satisfaction with the government’s vaccination campaign in relation to the perceived information of the symptoms and consequences of the HZ virus, those who felt satisfied or fairly satisfied with the vaccination campaign felt sufficiently informed about the symptoms and consequences of the virus (Chi-square = 26.847; DF = 15; *p*-value = 0.030) (Table 6).

(c) Perception of the Herpes Zoster vaccination

Regarding attitudes toward vaccination and perceptions of risk and protection, it appears that when asked, “I am in favor of Herpes Zoster vaccination”, 57.6% “totally agree”, 12.9% “partially agree”, 15.9% “partially disagree”, and 13.5% “totally disagree” (Table 7).

In general, frail patients were “ I am confident in the progress of science in the field of vaccines, particularly Herpes Zoster”, with 68.2% reporting they “totally agree” with the statement, and 23.5% stating they “partially agree”, while 5.3% “partially disagree”, and 2.9% “totally disagree”.

Concerning the statement “I believe the vaccine can protect me in the future”, it emerged that 65.3% “totally agree”, 28.8% “partially agree”, 5.3% “partially disagree”, and 0.6% “totally disagree”. The perception of protection is high concerning the future. Those with rheumatological diseases expressed a high percentage of agreement concerning the statement (50%, “totally agree”; 17.7%, “partially agree”; 25.0%, “totally disagree”; 8.3%, “partially disagree”) (Chi-square = 17.886; DF = 3; *p*-value = 0.001).

Considering the probability of contracting the virus if one does not receive the vaccine, based on the question “I think the probability of contracting Herpes Zoster is high if I do not get vaccinated”, it was found that 44.7% “totally agree”, while 39.4% “partially agree”, 10.6% “partially disagree”, and 5.3% “totally disagree”.

The perceived protection of the vaccine also extends to other people close to the respondents. It was found that 61.2% “totally agree” with the statement “I believe that by vaccinating myself I protect people close to me”, 30.6% “partially agree”, 5.3% “partially disagree”, and 2.9% “totally disagree”.

Regarding risk perception, it was shown that for the item “If I did not have other diseases, I would not get the Herpes Zoster vaccine”, the participants’ answers showed a similar distribution of agreement/disagreement. It was found that 24.1% “totally agree” with the statement”, 28.2% “partially agree”, 21.8% “partially disagree”, and 25.9% “totally disagree”.

This affirmation is especially true for those whose present diseases include hypertension (Chi-square = 8.331; DF = 3; *p*-value = 0.040). Concerning this affirmation, 47.6% stated they “partially agree”, 19.0% “totally agree”, 15.2% “partially disagree”, and 18.2% “totally disagree”.

The perceived utility of the vaccine extends to the whole population, not only to the frail. In fact, to the assertion “I believe that everyone should get the Herpes Zoster vaccine”, participants responded with 56.5% “totally agree”, 31.8% “partially agree”, 8.2% “partially disagree” and 3.5% “totally disagree” (Table 7).

The degree of dissatisfaction with the government’s vaccination campaigns increases among those who “think everyone should get the Herpes Zoster vaccine” (Chi-square = 33.040; DF = 15; *p*-value = 0.005), increasing to 60.0% for those who are “totally dissatisfied” and 69.2% for those who are “fairly dissatisfied” (Table 8).

## 4. Discussion

The exploratory analysis carried out on the anti-HZ vaccination campaign in a sample of frail adult patients opens up multiple reflections. Health behaviors enacted by people are closely linked to representations of illness, health, and prevention constructed through experience in their social and healthcare local settings [32,39,41,42,44].

In the case of the research being presented, it is possible to think of a core representation of the HZ and related vaccination, understood as a collective memory of the specific disease within Italian culture. (In Italian culture, for example, the Herpes Zoster virus is commonly called St. Anthony’s fire. Indeed, reference is made symbolically to the burns suffered by the saint as a result of clashes with the devil in the desert, which are associated with the intense burning caused by shingles) [45]. In the peripheral system, on the other hand, it is possible to discern features that contain elements related to the individual differences and personal experiences of frail adult patients realized with the disease and anti-HZ vaccination [26]. The implementation of vaccination campaigns should take these representations into account to achieve effective interventions, particularly when it comes to frail patients, who often suffer from complex, often multiple chronic diseases, with the presence of comorbidities, clinical instability, polypharmacy, and reduced self-sufficiency [14,21,44,46].

The present study stems from the following research questions: What directs these patients to receive anti-HZ vaccination? What are the cultural and individual models related to the representation of the disease, that is to say, the identity of the disease, its consequences, as well as the perceived sense of power regarding the adoption of effective treatments such as vaccines [43,47]? Finally, what sources of information are authoritative in the construction of these representations?

From the first dimension explored in our survey, concerning the knowledge and perception of HZ and its associated risks, it emerged that the fragile adult patients participating in the research have a representation of HZ as a serious disease, about whose symptoms and consequences they perceive themselves to be sufficiently informed, unlike other studies in which a lack of knowledge about the disease and vaccine emerges [21,29]. The participants, however, do not overly fear the effects of the disease on their health. We hypothesize that the little fear of the effects of the virus, as well as the good perceived knowledge of the virus, may be related to the methodology used in the research design. In other words, patients filled out the questionnaire after being recruited and informed about the virus and the vaccine immediately before vaccination by healthcare personnel who were already following them on the care pathway in the reference ward; thus, they were already adhering to a measure of protection from the virus itself. Women appear more fearful, while with advancing age, patients feel they are less aware of the symptoms and consequences of the virus. This hints at how health literacy needs to be sensitive to the plurality of targets it addresses [40], using more usable communication and communication channels, especially for older people, who in this case seem to perceive themselves as less informed about the virus [44,48,49].

The second dimension explored concerns the information process, as illness representations are influenced by the information held by the person, both new and previous [41,42]. Therefore, it becomes crucial to understand the influence that the information channels used, such as the press, social media, and health personnel, have in this individual and cultural construction process [20]. The sociological literature has pointed out that, for example, while the progressive development of new mass media has expanded the range of information available, it has also exposed the risk of confusing and contradictory information, as it is not always subjected to criteria of reliability and verifiability [50,51,52,53,54]. Social media networks have been cited as a source of misinformation by public health professionals themselves [55]. Misinformation about vaccines alters an individual’s perception of disease severity and vulnerability, affecting the calculation of the risk/benefit ratio and consequently increasing vaccine hesitancy [56,57,58,59,60,61].

Patients who participated in the research stated that they mainly used healthcare personnel as a source of information. In this regard, it is worth noting that the recruited patients were largely informed by the healthcare personnel who were already following them for prior chronic conditions, and this influenced the choice of the information channel used to access the information. People construct illness representations and accept healthcare interventions when they can actively construct information, which is then supplemented with prior experiences, as in this case with experiences of pre-existing conditions [31,32,33]. In the process of self-regulation, this aspect is most important for handling self-care behaviors and for fostering “intrinsic motivation” in sufferers, that is, the conscious and satisfactory enactment of behaviors most beneficial to their well-being [40].

Information seeking is an active process, and this is evidenced even more by the fact that it differs by gender and profession in our sample: the survey showed that women use mainly media (TV and radio), while teachers use mainly the web and social web. In general, those who perceive themselves to be fairly well informed about HZ and its symptoms are also satisfied with the vaccination information campaigns disseminated by the government; the least satisfied are those who have an education degree or higher. The percentage of satisfaction with government campaigns is 58.9%; however, the percentage of those in the dissatisfaction and neutral area remains high (41.1%). This last question will need to be explored further in subsequent research.

People trust mainly the information they receive from healthcare personnel [60,62] and family members. This is in line with studies that show how opinions and beliefs, which are peculiar to the social network to which one belongs, can have a major impact on an individual’s will and behavior, including in healthcare, influencing beliefs about vaccines [63,64,65,66]. All of this points to the need for greater involvement of a patient’s family members in care pathways and preventive actions, particularly in the case of frail patients, who, as they age, rely on the advice of family members while trusting health professionals. Sociology has long theorized how the strength of beliefs is associated with social feedback from significant others as opinions of family members, friends, and healthcare providers had a significant impact on the willingness to vaccinate [64,65,67].

Finally, the third dimension of this study focused on the perception of the HZ vaccine as a risk or resource for implementing preventive behaviors. Regarding the perception of vaccine risk and resources, as a tool for health empowerment, it emerges that these patients are favorable to the vaccine; they perceive its protection toward themselves and their loved ones; they also trust the science behind this new vaccine and consider it a risk to their already precarious health not to be vaccinated. They believe that an anti-HZ vaccine is a useful tool for all; this is in line with previous research on vaccination against COVID-19 [68,69]. However, a proportion of these frail adult patients, if they had no other pathologies, would not deem it necessary to have this vaccine. A percentage of them fear vaccine side effects nonetheless, in line with the literature [21]. It could be hypothesized that the fragile condition predisposes to increased alertness to possible side effects. It could also be assumed that since the adjuvanted recombinant vaccine is a newer vaccine, these patients are probably still wary of the side effects produced.

Finally, it should not be forgotten that these fragile patients have experienced the COVID-19 pandemic period, during which much health information, particularly about COVID-19 vaccination, was conveyed, including numerous no-vax currents. The constant acquisition of information alleviated anxiety in some individuals and motivated them to vaccination, while in others, it fueled doubts and misgivings about it [23,70,71,72,73].

In the present research, it was found that patients with hypertension were the ones who most expressed fear toward the side effects of the anti-HZ vaccine. The percentage of these patients who report being concerned about the side effects of the vaccine stands at 66.6%. This is probably because much information about COVID-19 vaccine side effects concerning this group of patients has been disseminated [74]. It can be speculated that this fear of side effects is a reflection of the cultural scenario in which COVID-19 vaccination is placed.

From these considerations, it becomes crucial to identify those factors that could direct the population in making their vaccination choice and consequently act so that vaccination promotion and immunization campaigns can have a greater impact; for example, by planning timely and targeted information campaigns to counter negative messages and misinformation which convey messages of fear, uncertainty, and skepticism [54,75]. On the other hand, these campaigns should reinforce feelings of confidence in vaccines, as well as feelings of individual and collective efficacy and responsibility [66].

### Limitations and Future Developments

A limitation of this research resides in the fact that the questionnaire carried out was only administered to frail adult patients who adhered to vaccination. It should also be extended to those who choose not to vaccinate, and those who do not have a fragile condition (a condition not present in our sample).

The second limitation concerns the location of the research, which should still be extended to other regional or national centers willing to adopt the same strategy of hospital ambulatory intervention for vaccine administration.

In terms of possible research developments, it might be useful to expand the administration of the prepared questionnaire to the caregiver group as well since individual positioning concerning care and prevention practices is influenced by cultural group membership, as evidenced in the literature [62,63,65]. An indication emerged to expand the research on HZ representations by conducting a pre-test and a post-test of the intervention to verify whether and how the vaccination campaign changed the representations. This would make it possible to plan future vaccination campaigns for different diseases. Such campaigns should be carried out through strategies and paradigms constructed based on the baseline sample’s knowledge of the disease and the individual and contextual behaviors that can promote vaccine adherence.

## 5. Conclusions

In conclusion, it emerges that vaccination campaigns (prevention, information, and awareness) should be designed not only based on the target audience but also by paying attention to users’ specific individual and contextual needs and representations regarding disease and health behaviors to implement effective preventive interventions that improve vaccine adherence.

Vaccination campaigns should take into account two levels that are crucial in the present research and the literature: the patient, health personnel, and caregiver relationship; and the dissemination of targeted and clear information through institutional communication channels. These include not only the traditional channels (TV, print, radio, etc.) but also web and social web platforms and additional online platforms that have developed in recent years.

In conclusion, we have additionally discussed a number of different theoretical perspectives and dimensions to highlight some of the possible conceptual directions when analyzing vaccination from public health, psychological, and sociological perspectives. The aim is to inspire new and innovative empirical, methodological, and theoretical research in the interdisciplinarity of vaccines, particularly in relation to risk and uncertainty, financial interests, power, and inequality. In doing so, we hope to influence analysts to engage with a range of new or under-explored questions within the field of vaccines, while also enabling scholars to find new ways of analyzing existing ones. There is good evidence that vaccine uptake is shaped by socio-structural forces. These forces not only produce different worldviews, but also present different vaccination opportunities and availability, promote different information and knowledge about vaccination, and create a set of priorities in managing a life at risk.

## Figures and Tables

**Table 1:** Socio-demographic factors and Herpes Zoster experience of total sample.

Main Categories	Variables		%
Socio-demographic factors	Gender	Men	62.3%
		Women	37.7%
	Marital status	Married	67.6%
		Separated/divorced	6.5%
		Single	19.4%
		Widowed	3.0%
		Cohabiting	3.5%
	Level of schooling	Secondary school degree	77.6%
		University degree/post-graduate training	22.4%
	Work	Office workers	14.1%
		Teachers	4.1%
		Homemakers	11.8%
		Laborer/craftsman	11.8%
		Self-employed	10.6%
		Student	2.4%
		Retiree	28.2%
		Unemployed	8.2%
		Other	8.2%
HZ infection		Yes	25.0%
		no	75.0%

**Table 2:** Frequency table of a multiple response set of previous pathologies.

Variable		Total Count%
Previous pathologies	Chronic heart disease	17.5%
	Hypertension	63.3%
	Diabetes mellitus	21.1%
	Pulmonary diseases	7.8%
	Immunosuppressive therapy	77.1%
	Rheumatological diseases	7.2%
	Oncological pathologies	6.6%
	Dialysis treatment	1.8%
	Organ transplantation	2.4%
	Hematopoietic stem cell transplantation	4.8%

**Table 3:** Percentage frequencies of responses to items on the HZ virus and risk perception.

Items on Knowledge of Pathology and Perception of Risk		%
I am sufficiently informed about the Herpes Zoster virus	Totally disagree	6.5%
	Partially disagree	8.8%
	Partially agree	41.8%
	Totally agree	42.9%
I am adequately informed about the symptoms and consequences of the Herpes Zoster virus	Totally disagree	5.9%
	Partially disagree	11.8%
	Partially agree	41.8%
	Totally agree	40.6%
Herpes zoster is a severe disease	Totally disagree	3.5%
	Partially disagree	10.0%
	Partially agree	44.7%
	Totally agree	41.8%
I do not fear the effects of Herpes Zoster	Totally disagree	23.5%
	Partially disagree	22.9%
	Partially agree	30.0%
	Totally agree	23.5%

**Table 4:** Frequency percentage of answers to items related to information sources and confidence in sources.

Items on Information Sources and Confidence in Sources		%
What are the major sources of information used?	Media (TV, radio)	10.0%
	Web and social web	14.7%
	Doctors	73.5%
	Word-of-mouth	1.8%
In your experience, how satisfied are you with the vaccine information and awareness campaigns implemented by the Italian government?	Totally dissatisfied	8.8%
	Somewhat dissatisfied	15.3%
	Neither satisfied nor dissatisfied	14.1%
	Fairly satisfied	31.8%
	Satisfied	27.1%
	I have had no experience with this	2.9%
I have confidence in the information I have received from the medical staff.	Totally disagree	3.5%
	Partially disagree	4.1%
	Partially agree	25.9%
	Totally agree	66.5%
I have confidence in the information I have acquired from the media.	Totally disagree	7.1%
	Partially disagree	20.0%
	Partially agree	45.9%
	Totally agree	27.1%
I have confidence in the information I have acquired from my family members.	Totally disagree	4.7%
	Partially disagree	8.2%
	Partially agree	42.4%
	Totally agree	44.7%

**Table 5:** Percentage frequencies for the item “information sources used” and the variables Gender and Occupation.

Informative Sources						
		Media (TV, Radio)	Web and Social Web	Doctors	Word-of-Mouth	*p*-Value
Gender	Women	17.2%	18.8%	64.1%	0.0%	0.023
	Men	5.7%	12.3%	79.2%	2.8%	
Work	Office worker	0.0%	29.2%	70.8%	0.0%	0.004
	Self-employed	5.6%	22.2%	72.2%	0.0%	
	Teacher	28.6%	42.9%	28.6%	0.0%	
	Homemakers	35.0%	10.0%	55.0%	0.0%	
	Laborer/craftsman	25.0%	0.0%	75.0%	0.0%	
	Student	25.0%	0.0%	75.0%	0.0%	
	Retiree	8.3%	12.5%	72.9%	6.3%	
	Unemployed	0.0%	6.7%	93.3%	0.0%	

**Table 6:** Percentage frequencies comparing the items “Satisfaction with government information campaigns” and “Information about the virus and its consequences”.

In Your Experience, How Satisfied Are You with the Vaccine Information and Awareness Campaigns Implemented by the Italian Government?								
		Totally	Somewhat Dissatisfied	Neither Satisfied Nor Dissatisfied	Fairly Satisfied	Satisfied	I Have Had No Experience with This	*p*-Value
I am sufficiently informed about the Herpes Zoster virus	Totally disagree	0.0%	7.7%	4.2%	9.3%	2.2%	40.0%	0.028
	Partially disagree	13.3%	15.4%	12.5%	9.3%	2.2%	0.0%	
	Partially agree	46.7%	34.6%	33.3%	53.7%	34.8%	40.0%	
	Totally agree	40.0%	42.3%	50.0%	27.8%	60.9%	20.0%	
	Tot.	100%	100%	100%	100%	100%	100%	
I am adequately informed about the symptoms and consequences of the Herpes Zoster virus	Totally disagree	6.7%	7.7%	0.0%	20.0%	4.3%	20.0%	0.030
	Partially disagree	22.3%	23.1%	20.8%	0.0%	2.2%	0.0%	
	Partially agree	23.0%	38.5%	45.8%	60.0%	32.6%	60.0%	
	Totally agree	49.0%	30.8%	33.3%	20.0%	60.9%	20.0%	
	Tot.	100%	100%	100%	100%	100%	100%	

**Table 7:** Frequency of answers to items on vaccine and risk perception.

Attitudes Toward Vaccine and Perceptions of Risk/Protection		%
I am in favor of Herpes Zoster vaccination	Totally disagree	13.5%
	Partially disagree	15.9%
	Partially agree	12.9%
	Totally agree	57.6%
I am confident in the progress of science in the field of vaccines, particularly Herpes Zoster	Totally disagree	2.9%
	Partially disagree	5.3%
	Partially agree	23.5%
	Totally agree	68.2%
I believe the vaccine can protect me in the future	Totally disagree	0.6%
	Partially disagree	5.3%
	Partially agree	28.8%
	Totally agree	65.3%
I think the probability of contracting Herpes Zoster is high if I do not get vaccinated	Totally disagree	5.3%
	Partially disagree	10.6%
	Partially agree	39.4%
	Totally agree	44.7%
I believe that by vaccinating myself I protect people close to me	Totally disagree	2.9%
	Partially disagree	5.3%
	Partially agree	30.6%
	Totally agree	61.2%
If I did not have other pathologies, I would not get the Herpes Zoster vaccine	Totally disagree	25.9%
	Partially disagree	21.8%
	Partially agree	28.2%
	Totally agree	24.1%
I worry about the side effects of the vaccine	Totally disagree	18.8%
	Partially disagree	18.2%
	Partially agree	42.9%
	Totally agree	20.0%
I believe that everyone should get the Herpes Zoster vaccine	Totally disagree	3.5%
	Partially disagree	8.2%
	Partially agree	31.8%
	Totally agree	56.5%

**Table 8:** Percentage frequencies between item “Satisfaction with government information campaigns” and the item “Perceived usefulness of the vaccine”.

In Your Experience, How Satisfied Are You with the Vaccine Information and Awareness Campaigns Implemented by the Italian Government?								
		Totally Dissatisfied	Somewhat Dissatisfied	Neither Satisfied Nor Dissatisfied	Fairly Satisfied	Satisfied	I Have Had No Experience with This	*p*-Value
I believe that everyone should get the Herpes Zoster vaccine	Totally disagree	0.0%	0.0%	0.0%	3.7%	4.3%	40.0%	0.005
	Partially disagree	13.3%	15.4%	16.7%	3.7%	4.3%	0.0%	
	Partially agree	26.7%	15.4%	29.2%	37.0%	39.1%	20.0%	
	Totally agree	60.0%	69.2%	54.2%	55.6%	52.2%	40.0%	
	Tot.	100%	100%	100%	100%	100%	100%	

## Data Availability

Written informed consent was obtained from the subjects to publish this paper. The archived data is not public and can be requested by writing to the corresponding author.
